# Can Cardiopulmonary Rehabilitation Facilitate Weaning of Extracorporeal Membrane Oxygenation (CaRe-ECMO)? Study Protocol for a Prospective Multidisciplinary Randomized Controlled Trial

**DOI:** 10.3389/fcvm.2021.779695

**Published:** 2022-01-07

**Authors:** Yu Zheng, Hao Sun, Yong Mei, Yongxia Gao, Jinru Lv, Dijia Pan, Lu Wang, Xintong Zhang, Deliang Hu, Feng Sun, Wei Li, Gang Zhang, Huazhong Zhang, Ying Chen, Shenrui Wang, Zhongman Zhang, Baoquan Li, Xufeng Chen, Jinsong Zhang, Xiao Lu

**Affiliations:** ^1^Department of Rehabilitation Medicine, The First Affiliated Hospital of Nanjing Medical University, Nanjing, China; ^2^Department of Emergency Medicine, The First Affiliated Hospital of Nanjing Medical University, Nanjing, China; ^3^Department of Rehabilitation Medicine, Qingdao Municipal Hospital, Qingdao, China

**Keywords:** cardiopulmonary rehabilitation, extracorporeal membrane oxygenation, weaning, multidisciplinary, randomized controlled trial, open-label

## Abstract

**Background:** Mortality of patients suffering from critical illness has been dramatically improved with advanced technological development of extracorporeal membrane oxygenation (ECMO) therapy. However, the majority of ECMO-supported patients failed to wean from ECMO therapy. As one of several options, cardiopulmonary rehabilitation serves as effective intervention in the improvement of cardiovascular and respiratory function in various major critical illness. Nonetheless, its role in facilitating ECMO weaning has not yet been explored. The purpose of this study is to investigate the effectiveness of cardiopulmonary rehabilitation on rate of ready for ECMO weaning in ECMO-supported patients (CaRe-ECMO).

**Methods:** The CaRe-ECMO trial is a randomized controlled, parallel group, clinical trial. This trial will be performed in a minimum number of 366 ECMO-supported eligible patients. Patients will be randomly assigned to either: (1) the CaRe-ECMO group, which will be treated with usual care including pharmacotherapy, non-pharmacotherapy, and specific nursing for ECMO therapy and the CaRe-ECMO program; or (2) the control group, which will receive usual care only. The CaRe-ECMO program consists of protocolized positioning, passive range of motion (PROM) training, neuromuscular electrical stimulation (NMES), surface electrical phrenic nerve stimulation (SEPNS), and pulmonary rehabilitation. The primary outcome of the CaRe-ECMO trial is the rate of ready for ECMO weaning at CaRe-ECMO day 7 (refers to 7 days after the CaRe-ECMO program initiation). Secondary outcomes include rate of ECMO and mechanical ventilation weaning, total length in day of ready for ECMO weaning, ECMO weaning and mechanical ventilation, all-cause mortality, rate of major post-ECMO complications, ECMO unit length of stay (LOS) and hospital LOS, total cost for hospitalization, cerebral performance category (CPC), activities of daily living (ADL), and health-related quality of life (HRQoL).

**Discussion:** The CaRe-ECMO is designed to answer the question “whether cardiopulmonary rehabilitation can facilitate weaning of ECMO (CaRe-ECMO).” Should the implementation of the CaRe-ECMO program result in superior primary and secondary outcomes as compared to the controls, specifically the add-on effects of cardiopulmonary rehabilitation to the routine ECMO practice for facilitating successful weaning, the CaRe-ECMO trial will offer an innovative treatment option for ECMO-supported patients and meaningfully impact on the standard care in ECMO therapy.

**Clinical Trial Registration:** ClinicalTrials.gov, identifier: NCT05035797.

## Introduction

Recent improvements in technology have created extra chances for lifesaving, i.e., using extracorporeal membrane oxygenation (ECMO) as a bridge to win over time for the body and medical strategies struggling with critical illness (1). Patients with acute cardiac, pulmonary, or cardiopulmonary failure who have failed conventional treatment appeared to benefit from ECMO therapy (2). However, this population is often exposed to prolonged immobilization, which may lead to multiple complications and impaired physical function (3). Shortening the sedative course and managing issues such as intensive care unit (ICU)-acquired weakness and critical illness-induced cardiopulmonary function impairment should take priority, as this can greatly impact the post-ECMO prognosis. In this context, attention toward enhancing ICU recovery, reducing complications, and improving functional prognosis has started to be shifted to ECMO-supported patients (4). Upon this perspective, early rehabilitation is recommended to be applied in this population.

The general aim of early rehabilitation for critical illness is to improve or maintain cardiopulmonary function, which encounters the requirement of ECMO-supported patients (5). There is a variety of early rehabilitation techniques that may benefit patients with critical illness (6). However, due to the huge difficulties in the application of early rehabilitation in ECMO-supported patients, only a few evidence has been published (4, 7). Considering their pathophysiological condition, regardless of venovenous ECMO (VV-ECMO) or venoarterial ECMO (VA-ECMO), cardiopulmonary rehabilitation is hypothesized to compensate cardiopulmonary function of patients and consequently speed up the recovery process after the emergency period (normally after 72 h) (8). For instance, cardiac output and maximal oxygen uptake were proven to increase after one subtype of cardiopulmonary rehabilitation in intubated, sedated patients confined to bed in the ICU (9).

Upon the above rationale, the upcoming question is that what benefits of cardiopulmonary rehabilitation can be delivered to ECMO-supported patients. For clinicians, ECMO weaning is particularly challenging and annual ECMO mortality has been reported to be approximately ranged 40–70% (10, 11). According to our experience, delayed ECMO weaning also demonstrated worse functional prognosis and impaired health-related quality of life (HRQoL). Previous studies have documented that rate of mechanical ventilation weaning was improved in patients received early rehabilitation intervention, while its effectiveness in ECMO weaning remains unclear (12). Indeed, the successful weaning of ECMO is complex and multifactorial (13). ECMO weaning at the earliest possible time would be expected to improve outcomes, reduce cost, and optimize functional prognosis (14). This inspires us to hypothesize that if the medical rationale is based on its assumed benefits on cardiac function and oxygenation, then cardiopulmonary rehabilitation may subsequently contribute to earlier weaning of ECMO. Our perspective is to provide solutions with the application of cardiopulmonary rehabilitation and convert the aforementioned physiological changes (e.g., increased cardiopulmonary function) into patient-oriented outcomes (e.g., earlier weaning of ECMO and increased rate of ECMO weaning).

Considering the limited evidence in this aspect, ongoing innovation must move from guessing to exploring the effectiveness of cardiopulmonary rehabilitation through an adequately powered trial. Therefore, we design this multidisciplinary randomized controlled trial (RCT) to answer the following clinical question—can cardiopulmonary rehabilitation facilitate weaning of ECMO (CaRe-ECMO)? The results of this trial may subsequently provide innovative treatment solution, specifically the add-on effects of cardiopulmonary rehabilitation to the routine clinical practice for further successful weaning of ECMO.

## Methods/Design

### Trial Design

The CaRe-ECMO trial is a pragmatic, multidisciplinary, randomized controlled, parallel group, clinical trial. Table 1 shows the overview of the trial registration information. The trial protocol was developed according to the Consolidated Standards of Reporting Trials (CONSORT) statements for pragmatic trials and non-pharmacological treatment interventions (15, 16).

**Table 1:** The WHO trial registration data set for the CaRe-ECMO trial.

**Data category**	**Information**
Primary registry and trial identifying number	ClinicalTrials.gov Registry number: NCT05035797
Date of registration in primary registry	01 September 2021
Secondary identifying numbers	N/A
Trial protocol version	Version 1
Source(s) of monetary or material support	Nanjing Municipal Science and Technology Bureau
Primary sponsor	Nanjing Municipal Science and Technology Bureau
Secondary sponsor	N/A
Contact for public queries	XL, luxiao1972@163.com; JSZ, zhangjso@njmu.edu.cn; XFC, cxfyx@njmu.edu.cn
Contact for scientific queries	XL, luxiao1972@163.com; JSZ, zhangjso@njmu.edu.cn; XFC, cxfyx@njmu.edu.cn
Public title	Can cardiopulmonary rehabilitation facilitate weaning of extracorporeal membrane oxygenation (CaRe-ECMO)? A prospective multidisciplinary randomized controlled trial
Scientific title	Impact of cardiopulmonary rehabilitation on weaning of extracorporeal membrane oxygenation (CaRe-ECMO): A prospective multidisciplinary randomized controlled clinical trial
Countries of recruitment	China
Health condition(s) or problem(s) studied	Rate of ECMO weaning in critical care patients supported with ECMO
Intervention(s)	Active comparator: Usual care, ECMO and cardiopulmonary rehabilitation
Key inclusion and exclusion criteria	Placebo comparator: Usual care and ECMO
	Ages eligible for study: aged 18yr or order
	Sexes eligible for study: both
	Accepts health volunteers: No
	Inclusion criteria: see “Eligibility and withdrawal criteria” section, and Tables 2, 3
	Exclusion criteria: see “Eligibility and withdrawal criteria” section, and Tables 2, 3
Study type	Type: Pragmatic, multidisciplinary, randomized controlled, parallel group, clinical trial
	Allocation: Simple randomization
	Intervention model: Parallel assignment
	Masking: Assessor, physician, data analyst, and statistician blinded
	Primary purpose: Prevention and improvement
	Phase: N/A
Date of first enrollment	Not yet started
Target sample size	366
Recruitment status	Not yet started
Primary outcome(s)	Rate of ready for ECMO weaning at CaRe-ECMO Day 7
Key secondary outcomes	Rate of ECMO weaning, total length of ready for ECMO weaning, total length of ECMO weaning, rate of mechanical ventilation weaning, total length of mechanical ventilation, all-cause mortality, rate of major post-ECMO complications, ECMO Unit LOS, total hospital LOS, total cost for hospitalization, CPC, ADL, and HRQoL

*CaRe-ECMO, cardiopulmonary rehabilitation on extracorporeal membrane oxygenation; LOS, length of stay; CPC, cerebral performance category; ADL, activities of daily living; HRQoL, health-related quality of life*.

### Trial Objectives

The primary objective of the CaRe-ECMO trial is to investigate the impact of cardiopulmonary rehabilitation combined with usual care on rate of ready for ECMO weaning at CaRe-ECMO day 7 (refers to 7 days after the CaRe-ECMO program initiation), when compared to usual care alone. Secondary objectives are to evaluate the effects of cardiopulmonary rehabilitation on rate of ECMO weaning, total length of ready for ECMO weaning, total length of ECMO weaning, rate of weaning of mechanical ventilation, total length of mechanical ventilation, all-cause mortality, rate of major post-ECMO complications, ICU length of stay (LOS), total hospital LOS, total cost for hospitalization, cerebral performance category (CPC), activities of daily living (ADL), and HRQoL.

### Ethics Statement

The CaRe-ECMO trial has been prospectively registered at the ClinicalTrials.gov (https://register.clinicaltrials.gov/): NCT05035797, 1 September, 2021. The trial protocol has been reviewed and approved by the Research Ethics Committee at the First Affiliated Hospital of Nanjing Medical University. In accordance with the Declaration of Helsinki of 1964 (revised in 2013), a written informed consent will be obtained from the legal guardians of all the enrolled patients (17).

### Trial Setting

The CaRe-ECMO trial will be mainly conducted in the Department of Emergency Medicine, the First Affiliated Hospital of Nanjing Medical University (a 3,700-bed primary referral hospital in Nanjing, Eastern China), who is capable to provide ECMO therapy for ~200 critical care patients annually. Clinicians from the Department of Emergency Medicine are required to have training certifications in ECMO therapy and at least 5-year therapeutic experience in management of critical care patients supported with ECMO. The Department of Rehabilitation Medicine will mainly be responsible for cardiopulmonary rehabilitation delivery. Clinicians from both the departments will collaborate on cardiopulmonary rehabilitation prescription. Therapists are required to have a certified degree in cardiopulmonary rehabilitation and at least 3-year therapeutic experience with critical care patients. The trial coordinating group will implement the CaRe-ECMO program guidelines and standard operation procedures (SOPs) for all the medical staffs via a kickoff meeting to be held in September 2021 (Nanjing, China).

### Eligibility and Withdrawal Criteria

The consolidated criteria for patient enrollment are those: (1) aged 18 years or older; (2) eligible for receiving ECMO (VV or VA) therapy; (3) with mechanical ventilation; (4) with stable condition and eligible for cardiopulmonary rehabilitation after 72 h of ECMO; (5) with no contraindications for cardiopulmonary rehabilitation; (6) not pregnant; (7) with a life expectancy of more than 3 days; (8) wean from ECMO therapy within the first 3 days before the initiation of the CaRe-ECMO program; (9) not use ECMO as a bridge to recovery or definitive treatment (e.g., lung transplantation or heart transplantation); (10) not enrolled in another trial previously; and (11) sign informed consent form by the guardian (18). According to previously published guidelines, the specific indications and contraindications for VV-ECMO and VA-ECMO are given in Tables 2, 3, respectively (19, 20).

**Table 2:** Indications and contraindications for VV-ECMO.

**Indications for VV-ECMO (one or more of the following)**
1. Hypoxemic respiratory failure (PaO_2_/FiO_2_ < 80 mmHg), after optimal medical management (FiO_2_ > 80%)
2. pH < 7.25 with PaCO_2_ ≥ 60 mmHg for more than 6 h, despite optimal conventional mechanical ventilation (respiratory rate of 35 bpm and P_plat_ ≤ 30 cmH_2_O)
3. Airway obstruction, unable to establish advanced airways
**Contraindications for VV-ECMO**
1. Irreversible non-cardiac organ failure limiting survival (e.g., severe anoxic brain injury or metastatic cancer)
2. Irreversible and incapacitating central nervous system pathology
3. Irreversible and incapacitating cardiovascular system pathology
4. Systemic bleeding
5. Mechanical ventilation for more than 7 days with P_plat_ > 30 cmH_2_O and FiO_2_ > 90%
6. Limited vascular access (severe peripheral arterial disease, extreme obesity, amputated limbs, among others)
7. Older age (increasing risk of death with increasing age)

*VV-ECMO, venovenous extracorporeal membrane oxygenation; PaO_2_, partial pressure of arterial oxygen; FiO_2_, fraction of inspired oxygen; PaCO_2_, partial pressure of arterial carbon dioxide; P_plat_, plateau pressure*.

**Table 3:** Indications and contraindications for VA-ECMO.

**Indications for VA-ECMO (one or more of the following)**
1. Rapidly deteriorating or severe cardiogenic shock (cardiac Index < 2.2 L/min/m^2^ with ^*^ VIS > 40) with LVEF < 35%
2. Rapidly deteriorating or severe cardiogenic shock (cardiac Index < 2.2 L/min/m^2^ with ^*^ VIS > 40) with LVEF of 35–55%, and severe mitral regurgitation or aortic stenosis
3. Two consecutive lactate values ≥4 mmol/L (with at least 30 min interval between sampling), with non-decreasing trend on steady dose of inotropes and/or vasopressors
4. Witnessed cardiopulmonary resuscitation for more than 10 min or intermittent ROSC but unable to maintain
**Contraindications for VA-ECMO**
1. Irreversible non-cardiac organ failure limiting survival (e.g., severe anoxic brain injury or metastatic cancer)
2. Irreversible and incapacitating central nervous system pathology
3. Irreversible and incapacitating cardiovascular system pathology
4. Systemic bleeding
5. Aortic dissection
6. Limited vascular access (severe peripheral arterial disease, extreme obesity, amputated limbs, among others)
7. Older age (increasing risk of death with increasing age)

*VA-ECMO, venoarterial extracorporeal membrane oxygenation; LVEF, left ventricular ejection fraction; SvO_2_, mixed venous oxygen saturation; ROSC, return of spontaneous circulation; VAD, ventricular assist device*.

* *Vasoactive-inotrope score (VIS): (Epinephrine dose + Norepinephrine dose) μg/kg/min × 100 + (Dopamine dose + Dobutamine dose) μg/kg/min*.

Patients will be withdrawn from the trial if: (1) the guardian makes such a request with no reasons; (2) the patient develops adverse events due to cardiopulmonary rehabilitation (e.g., rib fracture, limb fracture); and (3) the patient develops ECMO-related complications (e.g., bleeding, thromboembolism, severe infection) and is ever unsuitable for participating in the trial according to the point of view by clinicians.

### Enrollment, Randomization, and Allocation

Eligible patients who meet the eligibility criteria and are provided with ECMO therapy in our Department of Emergency Medicine will be enrolled in the CaRe-ECMO trial. Their guardians will receive a detailed explanation of trial purpose and procedures. The patients will have a same chance to be randomly assigned to the CaRe-ECMO group or the control group. They also will be informed that they have the right to withdraw from the trial at any time of the trial period.

**Figure 1:**
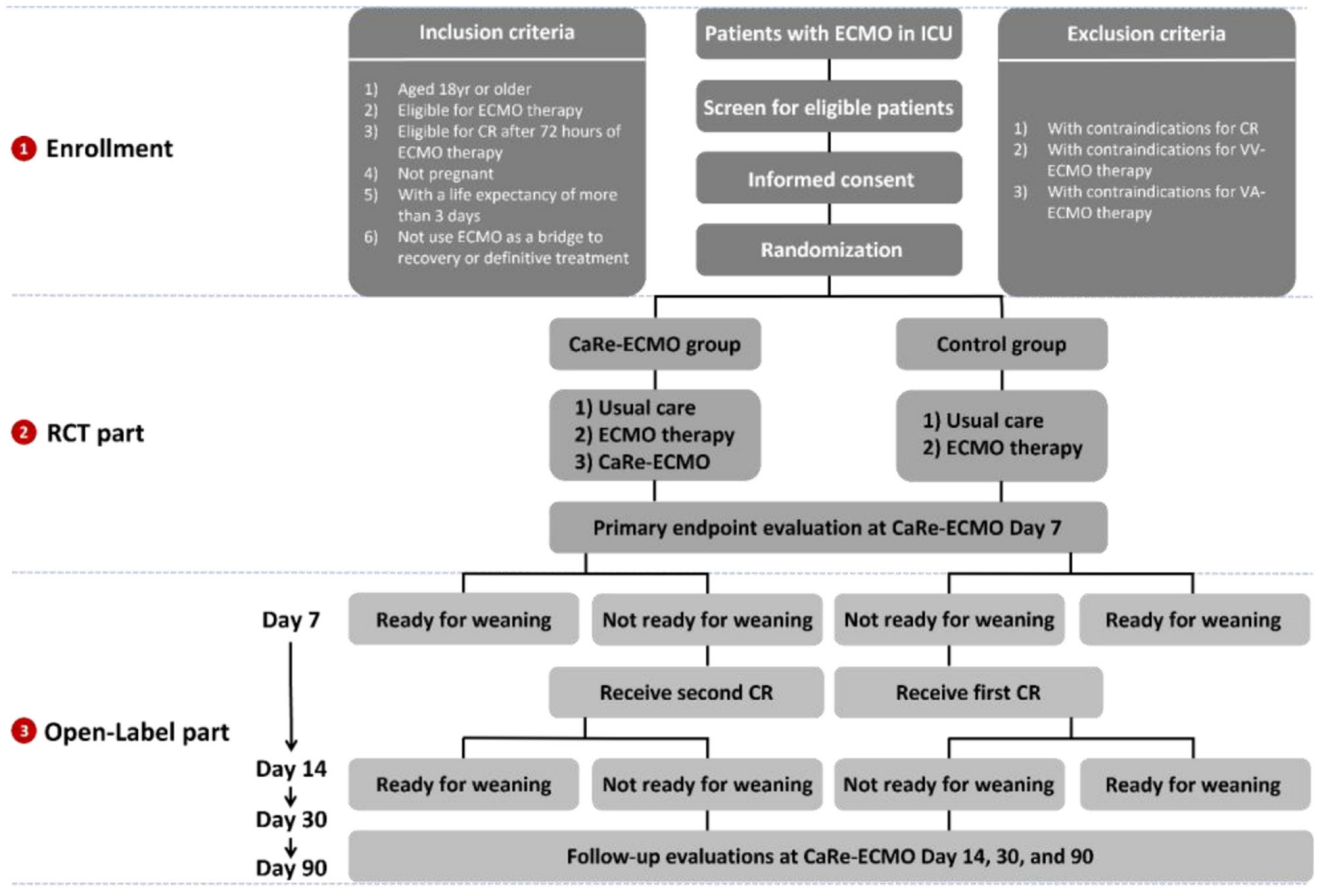
Overview of trial recruitment, randomization, and allocation. ECMO, extracorporeal membrane oxygenation; ICU, intensive care unit; CaRe-ECMO, cardiopulmonary rehabilitation on ECMO; CR, cardiopulmonary rehabilitation.

Simple randomization (1:1) will be applied using a computer generated random sequence, which will be independently administered and concealed by the Clinical Research Board from the School of Public Health of Nanjing Medical University (from here on called allocation center). Once a patient has been deemed eligible and the informed consent form has been signed, an independent trial assistant will register the participant, record her/his group allocation, and reveal the group allocation to those delivering the interventions (21). Figure 1 demonstrates the overview of the recruitment, randomization, and allocation based on the CONSORT principle.

### Interventions

#### CaRe-ECMO Group

**Figure 2:**
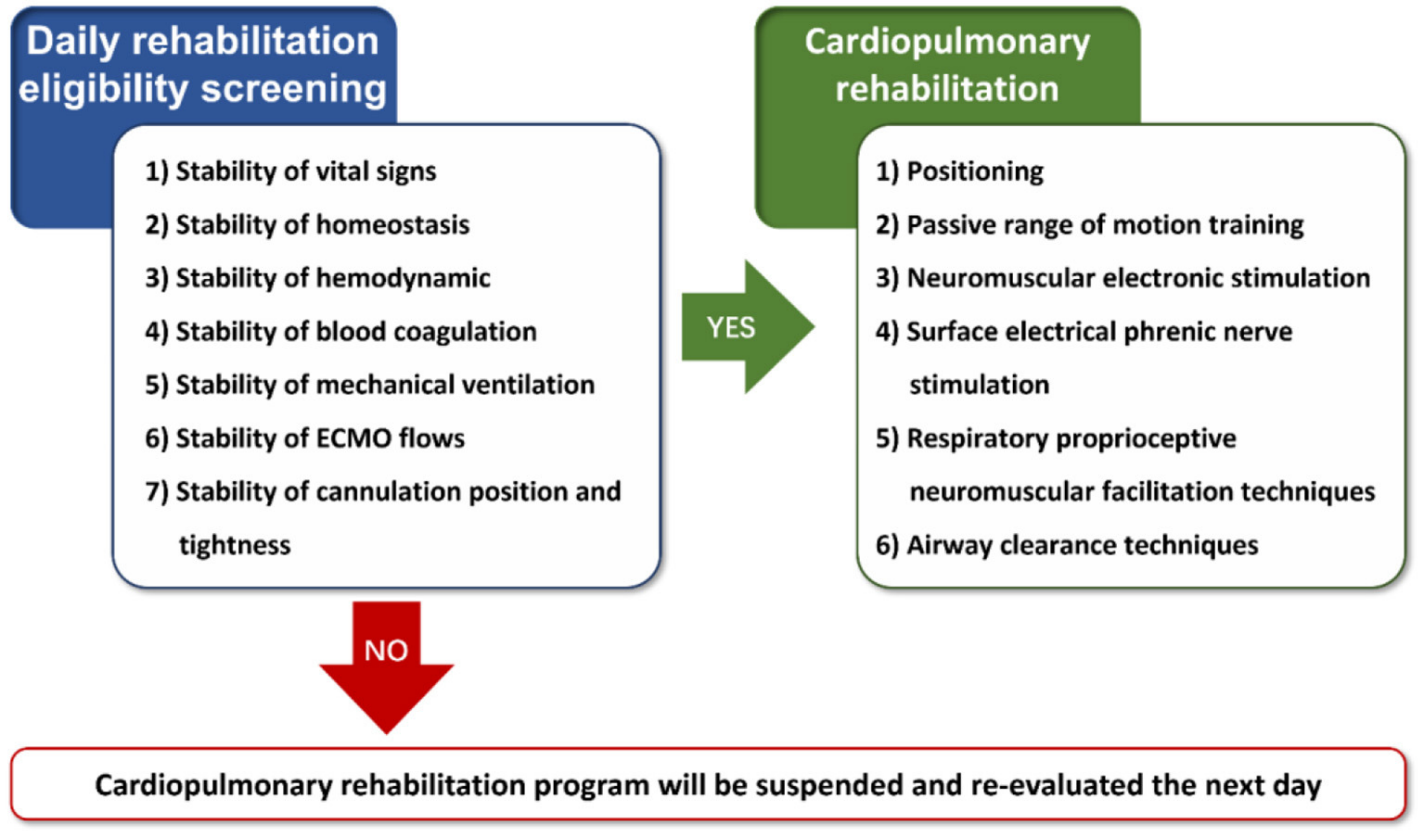
Eligibility screening flowchat for cardiopulmonary rehabilitation. ECMO, extracorporeal membrane oxygenation.

Patients in the CaRe-ECMO group will be treated with usual care, ECMO therapy, and cardiopulmonary rehabilitation program. Usual care normally comprises pharmacotherapy, mechanical ventilation, continuous renal replacement therapy (CRRT), intra-aortic balloon pump (IABP), and specific nursing for ECMO therapy and their original injuries, as appropriate. In addition, patients will receive cardiopulmonary rehabilitation from a multidisciplinary team (e.g., clinicians, rehabilitation physicians, nurses, and therapists) who were fully trained to provide care to every ECMO-supported patient when medically appropriate. A core group of clinicians and therapists (e.g., XL, ZS, XC, YZ, YC, and SW) are competent in the use of a screening procedure (e.g., stability of hemodynamic and vital signs, stability of blood coagulation, stability of homeostasis, stability of mechanical ventilation, stability of ECMO flows, and stability of cannulation position and tightness) to determine appropriateness and safety for cardiopulmonary rehabilitation delivery according to the experience from the University of Maryland Medical Center (4). Detailed daily eligibility screening flowchart for cardiopulmonary rehabilitation is shown in Figure 2. Once the patients pass the screen, the therapists will proceed to provide protocolized cardiopulmonary rehabilitation program, which encompasses five evidence-based components according to literature review: (1) positioning; (2) passive range of motion (PROM) training; (3) neuromuscular electrical stimulation (NMES); (4) surface electrical phrenic nerve stimulation (SEPNS); (5) respiratory proprioceptive neuromuscular facilitation (PNF) techniques; and (6) airway clearance techniques (22–24). Detailed content and protocol are given in Table 4. It should be emphasized that hemodynamic and vital signs, ECMO flows, and cannulation position and tightness will be continuously monitored during each rehabilitation session. In case any adverse events occur, cardiopulmonary rehabilitation session will be discontinued. Corresponding reasons for discontinuous and exact length of therapy will be recorded.

**Table 4:** Detailed content and protocol of cardiopulmonary rehabilitation.

**Cardiopulmonary rehabilitation**	**Components**	**Instructions**	**Duration and frequency**	**Rationale**
Positioning	Semi-reclining positioning (30°)	Nurse-assisted position change	Every 2 h	Improve oxygenation (25)
	Left or right lateral positioning (45°) Prone positioning			Improve ventilation/perfusion ratio of the lower part of lung (26) Improve oxygenation (27)
PROM training	PROM training for shoulder joint, elbow joint, wrist joint, hip joint, knee joint, and ankle joint	Therapist-assisted multidimensional movement of joints within normal ROM	20 min per session 1 session per day	1. Maintain joint mobility 2. Prevent contractures 3. Compress peripheral vessels to avoid thrombosis
NMES	NMES (Ruiyi S4, Nanjing Vishee Medical Technology Corporation, Nanjing, China) for triceps brachii, extensor carpal muscles, quadriceps, and tibialis anterior	1. Allocation of electrodes: surface electrodes attached to the motion points of muscles 2. Allocation confirmation: obtain a palpable muscle contraction of the targeted muscle 3. Stimulation parameters: pulse frequency of 40–60 Hz, pulse width of 0.2–0.4 ms, and rectangular, intermittent, bidirectional current	20 min per session 1 session per day	1. Maintain muscle mass, muscle strength 2. Prevent disuse atrophy 3. Compress peripheral vessels to avoid thrombosis
SEPNS	SEPNS (Diafun EDP, Arahelio Group, Guangzhou, China) for Diaphragm	1. Allocation of phrenic nerve: ultrasound scanning on supraclavicular region to identify phrenic nerve which is on the surface of anterior scalene muscle 2. Allocation confirmation: after the surface electrodes attachment, successful SEPNS can be confirmed with ultrasound imaging (M-mode) both at breathing and breath-hold phases reflecting changes of bilateral diaphragmatic thickness and mobility as compared to with no SEPNS 3. Stimulation principles and parameters: in synchrony with mechanical ventilation, pulse frequency of 40–60 Hz, pulse width of 0.2–0.4 ms, and rectangular, intermittent, bidirectional current	20 min per session 1 session per day	Prevent diaphragmatic ICU-acquired weakness
Respiratory PNF techniques	Manual thoracic expansion and compression	Therapist- or device-assisted pulmonary rehabilitation techniques	20 min per session 1 session per day	1. Improve chest wall compliance 2. Increase lung volume 3. Reduce and avoid atelectasis
Airway clearance techniques	Manual flutter mucus clearance	Therapist-, nurse- or device-assisted airway clearance techniques	20 min per session 1 session per day	1. Assist airway clearance 2. Reduce post-ECMO pulmonary complications (e.g., infection)
	High-frequency chest wall oscillation			
	Endotracheal suctioning			

*PROM, passive range of motion; NMES, neuromuscular electrical stimulation; SEPNS, surface electrical phrenic nerve stimulation; ICU, intensive care unit; PNF, proprioceptive neuromuscular facilitation*.

After randomized treatment, the primary outcome will be assessed at the CaRe-ECMO day 7 after last cardiopulmonary rehabilitation session. All the patients who are not responded (e.g., failure in fulfilling the criteria of ready for ECMO weaning and need continuous ECMO support), regardless of trial assignment, will be provided with open-label treatment of cardiopulmonary rehabilitation. Corresponding secondary outcomes will be assessed at the open-label stage according to the preplanned schedule.

**Figure 3:**
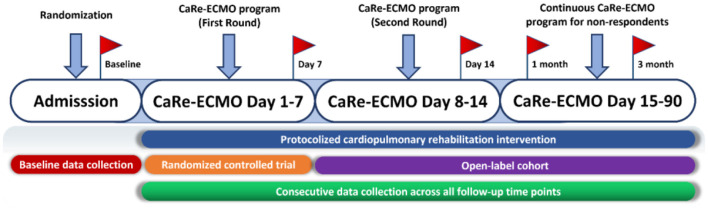
Flow diagram of the CaRe-ECMO program. First round of the CaRe-ECMO program refers to the protocolized cardiopulmonary rehabilitation delivery in the RCT part; second round of the CaRe-ECMO program refers to the protocolized cardiopulmonary rehabilitation delivery in the RCT part. Red flags symbolize measurement points of selective outcomes across all the follow-up time points. CaRe-ECMO, cardiopulmonary rehabilitation on extracorporeal membrane oxygenation; RCT, randomized controlled trial.

Detailed procedure of the CaRe-ECMO program is shown in Figure 3.

#### Control Group

No additional rehabilitation intervention, except usual care and ECMO therapy, will be provided to patients in the control group.

### Outcome Measures

#### Primary Outcome

The primary outcome of the CaRe-ECMO trial is the rate of ready for ECMO weaning at CaRe-ECMO day 7. It means that rate of ready for ECMO weaning will be calculated 7 days after cardiopulmonary rehabilitation delivery. Checklists to define ready for ECMO weaning (hereafter called ready for ECMO weaning checklists), either for VV-ECMO or VA-ECMO, have been prepared according to literature review and shown in Tables 5, 6 (20, 28). A fixed medical staff will be responsible for checkouts of listed items daily. Eligible patients defined as ready for ECMO weaning reveal the fulfillment of all the items in the checklists. Date of ready for ECMO weaning will be recorded and the rate of ready for ECMO weaning at CaRe-ECMO day 7 will be calculated.

**Table 5:** Ready for VV-ECMO weaning checklist.

**Ready for VV-ECMO weaning checklist**	
Move forward to the nest step when the former step is fulfilled, should all of the steps be fulfilled indication the patient is ready for ECMO weaning	
Step 1	The primary disease has been controlled or improved pulmonary function assessment: If VT < 6 mL/kg, FiO_2_ ≤ 60%, PEEP < 10 cmH_2_O, PaO_2_ > 70 mmHg, PaO_2_/FiO_2_ > 200 mmHg, PaCO_2_ < 45 mmHg, pH 7.35–7.45, improved changes in X-ray/CT scanning, lung compliance >0.8 ml/kg^*^cmH_2_O, then move to the next step
Step 2	Cardiac function assessment: If MAP > 70 mmHg, the heart rate increases over 20% of the baseline following the reduction of ECMO flow, then move to the next step
Step 3	Organ function and tissue perfusion assessment: If lactate < 2 mmol/L, SvO_2_ > 70%, no symptoms of respiratory distress and improved function of other damaged organs, then move to the next step
Step 4	Patient must tolerate a full weaning trial: If disconnect oxygen supply of the ECMO with no reduction of ECMO flow, observation for 6 h, monitoring the patient to maintain parameters mentioned in Step 1–3, then move to the next step
Step 5	If Step 1, 2, 3, and 4 are validated then the patient is under minimal ECMO support: Be sure that there is no CO_2_ retention and the patient can maintain normal oxygenation and normocarbia, and FiO_2_ reaches 21%, ECMO flow becomes 3–4L per minute and the sweep should be <1 L per minute, then move to the next step
Step 6	The patient is ready for VV-ECMO weaning

*VV-ECMO, venovenous extracorporeal membrane oxygenation; VT, tidal volume; FiO_2_, fraction of inspired oxygen; PEEP, positive end-expiratory pressure; PaO_2_, partial pressure of arterial oxygen; PaCO_2_, partial pressure of arterial carbon dioxide; MAP, mean arterial pressure; SvO_2_, mixed venous oxygen saturation*.

**Table 6:** Ready for VA-ECMO weaning checklist.

**Ready for VA-ECMO weaning checklist**	
Move forward to the nest step when the former step is fulfilled, should all of the steps be fulfilled indicating the patient is ready for ECMO weaning	
Step 1	The etiology of cardiac failure must be compatible with myocardial recovery
Step 2	Pulmonary function should not be severely impaired: If FiO_2_ ≤ 50%, PEEP < 10 cmH_2_O, PIP < 30 cmH_2_O, PaO_2_ > 70 mmHg, PaO_2_/FiO_2_ > 200 mmHg, PaCO_2_ < 45 mmHg, pH 7.35–7.45, no pulmonary edema in X-ray/CT scanning, then move to the next step
Step 3	Hemodynamic and cardiac functional assessment: If MAP > 70 mmHg and PPD > 30 mmHg for 24 h, the heart rate increases over 20% of the baseline following the reduction of ECMO flow, VIS of <10, LVEF of more than 40%, aortic VTI of more than 10 cm/s, decreasing trend of cTnT and BNP values, CVP < 12 cmH_2_O, lactate < 2 mmol/L, SvO_2_ > 70%, with improved function of other damaged organs, then move to the next step
Step 4	The patient must tolerate a full weaning trial: Increase ACT to 200 s, then gradually reduce ECMO flow to 66% for 15 min, and then to 33% of its baseline value for 15 min, and finally a minimum of 1–1.5 L/min for 15 min. During the weaning trial, monitoring the patient to maintain parameters mentioned in Step 3
Step 5	The patient is ready for VA-ECMO weaning

*VA-ECMO, venoarterial extracorporeal membrane oxygenation; FiO_2_, fraction of inspired oxygen; PEEP, positive end-expiratory pressure; PIP, peak inspiratory pressure; PaO_2_, partial pressure of arterial oxygen; PaCO_2_, partial pressure of arterial carbon dioxide; MAP, mean arterial pressure; PPD, pulse pressure difference; VIS, vasoactive-inotrope score; VTI, velocity time integral; cTnT, cardiac troponin T; CVP, central venous pressure; SvO_2_, mixed venous oxygen saturation; ACT, activated clotting time*.

#### Secondary Outcomes

Secondary outcomes are listed and elaborated as follows:

1) Rate of ECMO weaning will be calculated according to date of ECMO weaning fulfilled.
2) Total length of ready for ECMO weaning refers to exact length in day till patients fulfill all the criteria of ready for ECMO weaning according to daily checkout records (14).
3) Total length of ECMO weaning refers to exact length in day for patients treated with ECMO therapy (29).
4) Rate of mechanical ventilation weaning will be calculated according to date of mechanical ventilation weaning fulfilled. Daily screening of mechanical ventilation weaning will be strictly performed with checklist shown in Table 7 according to the American Thoracic Society and the American College of Chest Physicians Clinical Practice Guideline (30).
5) Total length of mechanical ventilation refers to exact length in day for patients treated with mechanical ventilation.
6) All-cause mortality is defined as rate of death due to any causes and will be calculated according to date of death (31).
7) Rate of major post-ECMO complications refers to rate of complications occurred after ECMO including but not limited to ECMO-related complications (e.g., thromboembolism), mechanical ventilation-related complications (e.g., pneumonia), newly developed myocardial infarction, acute kidney injury, neurologic events (e.g., stroke, seizures), and multiple organ failure (32). Information of major post-ECMO complications will be collected in the Complication Recording Sheet (Table 8).
8) Diaphragmatic thickness and mobility refer to ultrasound-guided evaluation of diaphragmatic thickness and mobility under M-mode (33).
9) Extracorporeal membrane oxygenation unit LOS accounts for length in day for stay of patients in the ICU unit.
10) Total hospital LOS accounts for total hospital LOS in day for stay of patients in both the ECMO unit and other departments.
11) Total cost for hospitalization will be calculated by addition of the cost of all the units and department admission.
12) Cerebral performance category will be recorded, for those successfully weaning of ECMO, to reflect post-ECMO neurological status (34).
13) Activities of daily living will be evaluated, for those successfully weaning of ECMO, with Katz Index (35).
14) Health-related quality of life will be measured, for those successfully weaning of ECMO, with SF-12 (36).

**Table 7:** Mechanical ventilation weaning checklist.

**Mechanical ventilation weaning checklist**	
• Ready for mechanical ventilation weaning: Daily check prerequisite of primary disease controlled; FiO_2_ ≤ 0.6, PaO_2_/FiO_2_ > 200 with PEEP ≤ 5 cmH_2_O; no or low dose vasopressor being used; continuous intravenous sedation minimized as appropriate; response to simple questions; cough during tracheal aspirations • Should the above ready for mechanical ventilation weaning criteria fulfilled, move through the following steps when the former step is fulfilled. Should all of the steps be fulfilled indicating weaning of mechanical ventilation	
Step 1	Weaning test
	• Gradually decrease PEEP to 5 cmH_2_O with possible increase of FiO_2_ to 0.6 (over 20–30 min)
	• If: desaturation occur (SpO_2_ < 88%) and persist (>5 min) during this trial, then fail
	• If not: blood gases determined 10–20 min after establishing PEEP at 5 cmH_2_O
	• If: under these conditions (PEEP = 5 cmH_2_O and FiO_2_ at 30–60%), PaO_2_/FiO_2_ < 200, then fail
	• If not: weaning test passes, forward through the next step within 24 h
Step 2	Prolonged weaning test
	• Mechanical ventilation mode of volume-assist control ventilation or pressure support
	• Mechanical ventilation parameters of VT < 10 mL/kg; P_plat_ or pressure support < 30 cmH_2_O; RR ≤ 35/min; PEEP = 5 cmH_2_O; FiO_2_ ≤ 50%
	• If: desaturation occur (SpO_2_ < 88%) and persist (>15 min) during this trial, then failed
	• If not: prolonged weaning test passes, forward through the next step within 24 h
Step 3	Spontaneous breathing trial
	• T piece or PSV with pressure support at +7 cmH_2_O
Step 4	Weaning of mechanical ventilation

*PEEP, positive end-expiratory pressure; FiO_2_, fraction of inspired oxygen; SpO_2_, oxygen saturation; PaO_2_, partial pressure of arterial oxygen; VT, tidal volume; P_plat_, plateau pressure; RR, respiratory rate; PSV, pressure support ventilation*.

**Table 8:** Complication recording sheet.

**Complication recording sheet**
Name: ___ Gender: ___ Age: ___ Inpatient ID: ___ Bed number:___ Date of record: ___ / ___ / ___, ___: ___ Main diagnosis:
**Neurological complications** **□ No □ Yes (If yes, then fill the following items)**
Date and time: ___ / ___ / ___, ___: ___
□ Cerebral hemorrhage □ Cerebral infarction □ Epileptic seizure
**Cardiovascular complications** **□ No □ Yes (If yes, then fill the following items)**
Date and time: ___ / ___ / ___, ___: ___
□ Cardiac arrest □ Newly developed myocardial infarction □ Heart failure □ Myocardium stunning (Ultrasound) □ Newly developed hypertension requiring vasodilator therapy □ Shock (□ Cardiogenic □ Hypovolemic □ Obstructive □ Distributive) □ North-south syndrome □ Tamponade (□ Blood □ Plasma □ Air)
**Respiratory complications** **□ No □ Yes (If yes, then fill the following items)**
Date and time: ___ / ___ / ___, ___: ___
□ Pneumothorax □ Pulmonary infection □ Pulmonary edema □ Pulmonary atelectasis □ Ventilator-associated lung injury
**Other complications** **□ No □ Yes (If yes, then fill the following items)**
Date and time: ___ / ___ / ___, ___: ___
Extremities complications: □ Distal ischemia □ Necrosis □ Deep venous thrombosis □ Osteofascial compartment syndrome □ Limb amputation Infection: Infection pathogen: □ G^+^ bacteria □ G^−^ bacteria □ Mycobacteria □ Fungus □ Virus and prion □ Protozoon Infection site: □ Blood □ Bone marrow □ Cerebrospinal fluid □ Ascites □ Hydrothorax □ Respiratory tract □ Skin, muscle, soft tissue □ Feces □ Urine □ Surgical site □ Wound site □ Other______ Bleeding: □ Cerebral hemorrhage □ Gastrointestinal bleeding □ Respiratory tract bleeding □ Cannulation site bleeding □ Thrombocytopenia □ Surgical site bleeding □ Hemolysis □ DIC □ Other______ Thrombosis: □ Pulmonary thromboembolism □ ECMO catheter thrombosis □ Limb thrombosis □ Mesenteric thrombosis □ Other______
**ECMO-related complications** **□ No □ Yes (If yes, then fill the following items)**
Date and time: ___ / ___ / ___, ___: ___
□ Oxygenator failure □ Raceway rupture □ Crack in connectors □ Heat exchanger malfunction □ Cannula problems □ Clots or thrombosis of circuit component □ Pump failure □ Clots hemofilter □ Circuit change □ Other______

*ID, identified number; DIC, disseminated intravascular coagulation; ECMO, extracorporeal membrane oxygenation*.

### Data Collection

Data in terms of rate of ready for ECMO weaning, rate of ECMO weaning, rate of mechanical ventilation weaning, all-cause mortality, and rate of major post-ECMO complications will be calculated throughout the index hospitalization and follow-up period (e.g., CaRe-ECMO days 7, 14, 30, and 90) according to daily evaluations recorded in the medical records. Outcomes, for instance ready for ECMO weaning, will be treated as time-to-event outcomes; therefore, date and timing of events will be also recorded to facilitate statistical analysis. Total length of ready for ECMO weaning, total length in hour of ECMO weaning, total length in day of mechanical ventilation, ECMO unit LOS, and total hospital LOS will be collected according to the medical records. CPC index, ADL score, and HRQoL score will be selectively collected among those successfully weaning of ECMO across discharge day (discharge from ECMO unit) and all follow-up time points (e.g., CaRe-ECMO days 30 and 90). In addition, diaphragmatic thickness and mobility will be evaluated with ultrasound (M-mode) every 3 days for determining the allocation of phrenic nerve and the efficacy of SEPNS. The detailed data collection schedule is given in Table 9.

**Table 9:** Scheduled events and timeline of the CaRe-ECMO trial.

		**Study period**						
		**Eligibility**	**Allocation**	**Post-Allocation**			**Close-out**	
	**Timepoint**	***–t_**1**_***	***t_**0**_***	***t_**1**_***	***t_**2**_***	***t_**3**_***	***t_**4**_***	***t_***x***_***
		**Day of ECMO**		**7 days after CaRe-ECMO**	**14 days after CaRe-ECMO**	**30 days after CaRe-ECMO**	**90 days after CaRe-ECMO**	Discharge day^*^
Enrollment	Eligibility check	X						
	Informed consent	X						
	Randomization		X					
Assignment	CaRe-ECMO group		X					
	Control group		X					
Variables	Demographics		X					
	Clinical characteristics		X					
	Rate of ready for ECMO weaning			X	X	X	X	
	Rate of ECMO weaning			X	X	X	X	
	Total length of ready for ECMO weaning	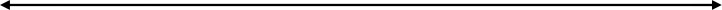						
	Total length of ECMO weaning	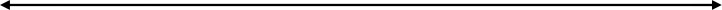						
	Rate of mechanical ventilation weaning			X	X	X	X	
	Total length of mechanical ventilation	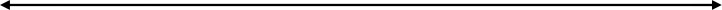						
	All-Cause mortality			X	X	X	X	
	Rate of major post-ECMO complications			X	X	X	X	
	Diaphragmatic thickness and mobility							
	ECMO unit LOS							X
	Total hospital LOS	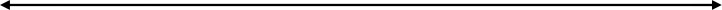						
	Total cost for hospitalization	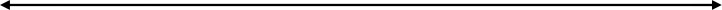						
	CPC			X	X	X	X	X
	ADL			X	X	X	X	X
	HRQoL			X	X	X	X	X

*CaRe-ECMO, cardiopulmonary rehabilitation on extracorporeal membrane oxygenation; LOS, length of stay; CPC, cerebral performance category; ADL, activities of daily living; HRQoL, health-related quality of life*.

* *Discharge day refers to discharge from ECMO unit and it may variate according to recovery process of patients*.

Baseline demographics and clinical characteristics, directly from the medical files at randomization, will also be collected including but not limited to the following information: age, gender, main diagnosis, comorbidities (diabetes mellitus, hypertension, respiratory infection, or others), the Acute Physiology and Chronic Health Evaluation II (APACHE II) score, ECMO mode (VV-ECMO or VA-ECMO), length in hour since mechanical ventilation, partial pressure of arterial oxygen/fraction of inspired oxygen (PaO_2_/FiO_2_) in mm Hg, positive end-expiratory pressure (PEEP), tidal volume in ml/kg of predicted body weight, respiratory rate in breaths/min, plateau pressure in cm of water, driving pressure in cm of water, arterial blood pH, PaO_2_ in mm Hg, partial pressure of arterial carbon dioxide (PaCO_2_) in mm Hg, N-terminal pro-B-type natriuretic peptide (NT-proBNP) in pg/ml, creatine kinase-MB (CK-MB) in ng/l, cardiac troponin T (cTnT) in ng/l, and ejection fraction (EF) in percentage. The above data will be recorded in the Baseline Data Collection Sheet (Table 10). Apart from that, daily clinical monitoring for both the condition and ECMO running status of patient will be performed. The detailed information will be recorded in the Daily Monitoring Sheet (Table 11).

**Table 10:** Baseline data collection sheet.

**Baseline data collection sheet**					
**Basic information**					
Name		Gender		Age	
Height (cm)		Weight (kg)		Bed number	
Inpatient ID		Phone number		BMI (kg/m^2^)	
Main diagnosis		APACHE II score		SOPA score	
Date of admission	___ / ___ / ___, ___: ___		Date of ECMO admission		___ / ___ / ___, ___: ___
Date of record	___ / ___ / ___, ___: ___				
**Medical history**					
Cardiovascular system	□ No □ Yes □ Cardiac surgery □ Cardiac intervention □ Myocardial infarction: □ Non ST-segment elevation □ ST-segment elevation □ Angina pectoris: □ Stable □ Unstable □ Structural heart disease □ Heart failure (□ New York Heart Association: □ I □ II □ III □ IV) □ Atrial fibrillation □ Hypertension				
Respiratory system	□ No □ Yes □ COPD □ Asthma □ Tuberculosis				
Cerebrovascular event and sequelae	□ No □ Yes □ Cerebral hemorrhage □ Cerebral infarction □ Dyskinesia □ Dysphagia □ Cognitive disorder □ Other______				
Diabetes	□ No □ Yes □ Type 1 diabetic mellitus □ Type 2 diabetic mellitus □ Impaired glucose tolerance				
Malignant tumor	□ No □ Yes □ Solid tumor □ Metastatic tumor □ Leukemia □ Lymphoma □ Other______				
Organ transplantation	□ No □ Yes				
Immune system disease	□ No □ Yes				
Medication history	□ Antiplatelet drugs: □ Aspirin □ Clopidogrel □ Dipyridamole □ Other______ □ Anticoagulants: □ Warfarin □ Dabigatran □ Rivaroxaban □ Heparin □ Other______ □ Antihyperlipidemic drugs: □ Statin □ Fibrates □ PCSK9 □ Other______ □ Antihypertensive drug: □β-receptor blocker □ ACEI □ ARB □ CCB □ Other______ □ Hypoglycemic drugs: □ Insulin □ Sulphonylurea □ Metformin □ Other______ □ Other______				
Personal history	□ Smoking history: Smoking index______ (Smoking index is a number of cigarettes smoked per day ^*^ years of tobacco smoking) □ History of alcoholism: Drinking index______g/day □ Dust and toxic exposure history				
Allergic history	□ No □ Yes □ Food □ Drug______				
Family history	□ No □ Yes □ Cardiovascular disease □ Cerebral disease □ Immune disease □ Endocrine disease □ Malignant tumor □ Other______				
**ECMO-Related information**					
Venue of ECMO operation	□ Out-of-hospital □ In-hospital				
ECMO cannulation	Drainage cannula □ Left □ Right Site: □ Femoral vein □ Axilla vein □ Internal jugular vein □ Right atrium Perfusion cannula □ Left □ Right Site: □ Femoral vein or femoral artery□ Axilla vein □ Internal jugular vein Whether additional drainage cannula is needed □ No □ Yes				
**Running status of ECMO**					
Indication	□ Cardiac □ Pulmonary □ ECPR				
ECMO type	□ VA-ECMO □ VV-ECMO □ Other______				
ECMO cannulation sites	VV-ECMO: □ No □ Yes (If yes, then fill the following items) □ Right femoral vein □ Left femoral vein □ Right internal jugular vein □ Left internal jugular vein □ Other______ VA-ECMO: □ No □ Yes (If yes, then fill the following items) □ Right femoral vein □ Left femoral vein □ Right internal jugular vein □ Left internal jugular vein □ Right femoral artery □ Left femoral artery □ Other______				
Distal collateral circulation	□ No □ Yes				
ECMO flow	ECMO blood flow______L/min ECMO gas flow______L/min ECMO FiO_2_______%				
Length from ECMO response to operation	______mins				
Length of ECMO operation	______mins				
Length of MV before ECMO	______days ______hours ______mins				
**First examination after ECMO**					
Blood routine examination	WBC______10^9^/L NE______% Hb______g/L PLT______10^9^/L				
Arterial blood gases	FiO_2_______% pH______ PO_2_______mmHg PCO_2_______mmHg BE______mmol/L ${\text{HCO}}_{3}^{-}$______mmol/L Lactate______mmol/l				
Inflammatory factors	C-reactive protein______mg/L Procalcitonin______ug/L Tumor necrosis factor______pg/mL IL-6______pg/mL Serum amyloid A______mg/L				
Biochemical examination	ALT______U/L AST______U/L TBIL______μmol/L DBIL______μmol/L ALP______U/L ALB______g/L BUN______mmol/L Cr______μmol/L Na^+^______mmol/L K^+^______mmol/L Cl^−^______mmol/L Ca^2+^______mmol/L				
Anticoagulation monitoring	Loading dose of heparin______IU/kg Maintenance dose of heparin______IU/kg APTT______s PT______s D-Dimer______ng/mL				
Antibiotic prophylaxis	□ No □ Yes______				
Cardiac function and myocardial injury markers	NT-proBNP______pg/ml cTnT______ng/L MyO______ng/L CK-MB______ng/L				
Electrocardiogram	□ Sinus arrhythmia □ Atrial arrhythmia □ Ventricular arrhythmia □ Supraventricular arrhythmia □ Junctional rhythm □ Other______ Ventricular rate______bpm				
Radiology	□ No □ Yes □ Pulmonary infection □ Pneumothorax □ Pulmonary edema □ Pulmonary atelectasis □ Pleural effusion/Hemothorax □ Cardiomegaly □ Pericardial effusion/Hemopericardium □ Cerebral edema □ Cerebral hemorrhage □ Ascites/Hemoperitoneum □ Ileus □ Other______				
Echocardiogram	EF______% VTI______ LVID______mm RVID______mm Valve □ Normal □ Abnormal □ Aortic valve (□ Moderate to severe aortic stenosis □ Moderate to severe aortic insufficiency) □ Mitral valve (□ Moderate to severe mitral stenosis □ Moderate to severe mitral insufficiency) □ Tricuspid valve (□ Moderate to severe tricuspid stenosis □ Moderate to severe tricuspid insufficiency) Structural heart disease □ No □ Yes □ Atrial septal defect □ Ventricular septal defect □ Tetralogy of fallot □ Endocardial cushion defect □ Single ventricle □ Other______ Pulmonary arterial hypertension □ No □ Yes Ventricular aneurysm □ No □ Yes				
Coronary angiography	□ No □ Yes Date of examination___ / ___ / ___ (If yes, then fill the following items) Stenosis □ No □ Yes Stenosis degree: I ≤ 25% II 26%-50% III 51%-74% IV ≥ 75% Left main artery: □ I □ II □ III □ IV Left anterior descending artery: □ I □ II □ III □ IV Left circumflex artery: □ I □ II □ III □ IV Right coronary artery: □ I □ II □ III □ IV Other______: □ I □ II □ III □ IV				
**Cardiac arrest (If ECPR, then fill the following items)**					
Venue of cardiac arrest	□ Out-of-hospital □ In-hospital				
Primary disease	□ Pulmonary □ Cardiac □ Anesthesia □ Other______				
Initial heart rhythm (Before CPR)	□ Ventricular fibrillation □ Pulseless ventricular tachycardia □ Pulseless electrical activity □ Cardiac standstill □ Other______				
Cardioversion or defibrillation	□ No □ Yes □ Unclear (If yes, then fill the following items) Drugs for cardioversion: □ Lidocaine □ Amiodarone □ Other______				
Length from cardiac arrest to CPR	Time of cardiac arrest___ / ___ / ___, ___: ___ Time of CPR___ / ___ / ___, ___: ___ Length______mins				
Length from CPR to ECPR	Time of CPR___ / ___ / ___, ___: ___ Time of ECPR___ / ___ / ___, ___: ___ Length______mins				

*ID, identified number; BMI, body mass index; ECMO, extracorporeal membrane oxygenation; APACHE, Acute Physiology and Chronic Health Evaluation; SOFA, Sequential Organ Failure Assessment; ECPR, external cardiopulmonary resuscitation; VV-ECMO, venovenous ECMO; VA-ECMO, venoarterial ECMO; FiO_2_, fraction of inspired oxygen; BE, base excess; MV, mechanical ventilation; CPR, cardiopulmonary resuscitation; ACEI, angiotensin-converting enzyme inhibitor; ARB, angiotensin receptor blocker; CCB, calcium channel blocker; PCSK9, proprotein convertase subtilisin/kexin type 9; COPD, chronic obstructive pulmonary disease; WBC, white blood cell; NE, neutrophil; Hb, hemoglobin; PLT, platelet; pO_2_, partial pressure of oxygen; pCO_2_, partial pressure of carbon dioxide; IL-6, interleukin-6; ALT, alanine aminotransferase; AST, aspartate aminotransferase; TBIL, total bilirubin; DBIL, direct bilirubin; ALP, alkaline phosphatase; AlB, albumin; BUN, blood urea nitrogen; Cr, creatinine; APTT, activated partial thromboplastin time; PT, prothrombin time; ACT, activated clotting time; NT-proBNP, N-terminal pro-B-type natriuretic peptide; cTnT, cardiac troponin T; MyO, myoglobin; CK-MB, creatine kinase-MB; EF, ejection fraction; VTI, velocity-time integral; LVID, left ventricular internal dimension; RVID, right ventricular internal dimension*.

**Table 11:** Daily monitoring sheet.

**Daily monitoring sheet**							
**Name: ___ Gender: ___ Age: ___ Inpatient ID: ___ Bed number: ___** **Date of record: ___ / ___ / ___, ___: ___ Main diagnosis:**							
**Evaluation**	**Index**	**CaRe-ECMO Day 1**	**CaRe-ECMO Day 2**	**CaRe-ECMO Day 3**	**CaRe-ECMO Day 4**	**…**	**CaRe-ECMO Day X**
Consciousness	RASS score						
	GCS score						
	BIS index						
	NSE						
	S100-β protein						
	EEG						
Temperature	Bladder temperature (°C)						
	Axillary temperature (°C)						
Fluid intake and output	Intake (ml)						
	Output (ml)						
	Output excess (ml)						
Nutrition	Enteral nutrition (Y/N)						
	Parenteral nutrition (Y/N)						
	Total calories (Kcal)						
Blood routine examination	WBC (10^9^/L)						
	NE (%)						
	Hb (g/L)						
	PLT (10^9^/L)						
Inflammatory factors	C-reactive protein (mg/L)						
	Procalcitonin (ug/L)						
	Tumor necrosis factor (pg/mL)						
	IL-6 (pg/mL)						
	Serum amyloid A (mg/L)						
Biochemical examination	ALT (U/L)						
	AST (U/L)						
	TBIL (μmol/L)						
	DBIL (μmol/L)						
	ALP (U/L)						
	ALB (g/L)						
	BUN (mmol/L)						
	Cr (μmol/L)						
	Na^+^ (mmol/L)						
	K^+^ (mmol/L)						
	Cl^−^ (mmol/L)						
	Ca^2+^ (mmol/L)						
Arterial blood gases	FiO_2_ (%)						
	pH						
	PO_2_ (mmHg)						
	PCO_2_ (mmHg)						
	BE (mmol/l)						
	${\text{HCO}}_{3}^{-}$ (mmol/l)						
	Lactate (mmol/l)						
Anticoagulation monitoring	Maintenance dose of heparin (IU/kg)						
	APTT (s)						
	PT (s)						
	D-Dimer (ng/ml)						
	ACT (s)						
Cardiac function and myocardial injury markers	NT-proBNP (pg/ml)						
	cTnT (ng/L)						
	MyO (ng/L)						
	CK-MB (ng/L)						
Electrocardiogram	Heart rhythm						
	Ventricular rate (bpm)						
Echocardiogram	EF (%)						
	VTI						
	LVID (mm)						
	RVID (mm)						
Hemodynamics	Vasoactive agent (Y/N)						
	Type of vasoactive agent						
	Dose of vasoactive agent						
	Vasoactive inotropic score						
	Systolic blood pressure (mmHg)						
	Mean arterial pressure (mmHg)						
	Central venous pressure (mmHg)						
	Inferior vena cava (mm)						
	Inferior vena cava variability (%)						
Ventilator parameters	Mechanical ventilation (Y/N)						
	Mode						
	Tidal volume (ml)						
	Respiratory rate (bpm)						
	FiO_2_ (%)						
	PEEP/CPAP (mmHg)						
	PIP (mmHg)						
	P_plat_ (mmHg)						
	Minute ventilation (L/min)						
	Pulmonary compliance (ml/cmH_2_O)						
	Inspiratory resistance						
	Expiratory resistance						
Radiology	Pulmonary infection						
	Pneumothorax						
	Pulmonary edema						
	Pulmonary atelectasis						
	Pleural effusion						
	Cardiomegaly						
	Pericardial effusion						
	Cerebral edema						
	Cerebral hemorrhage						
	Ascites						
	Ileus						
Pathogenic microorganism	Bacteria (Y/N)						
	Fungus (Y/N)						
	Virus (Y/N)						
	Location of the microorganism						
	Name of the microorganism						
Running status of ECMO	ECMO mode						
	ECMO blood flow						
	ECMO gas flow						
	ECMO FiO_2_ (%)						
Adjuvant therapy	CRRT (Y/N)						
	IABP (Y/N)						
	Other (Y/N)						

*ID, identified number; ECMO, extracorporeal membrane oxygenation; RASS, Richmond Agitation Sedation Scale; GCS, Glasgow Coma Scale; BIS, bispectral index; NSE, neuron-specific enolase; EEG, electroencephalography; WBC, white blood cell; NE, neutrophils; Hb, hemoglobin; PLT, platelet; IL-6, interleukin-6; ALT, alanine aminotransferase; AST, aspartate aminotransferase; TBIL, total bilirubin; DBIL, direct bilirubin; ALP, alkaline phosphatase; ALB, albumin; BUN, blood urea nitrogen; BE, base excess; Cr, creatinine; FiO_2_, fraction of inspired oxygen; pO_v2_, partial pressure of oxygen; pCO_2_, partial pressure of carbon dioxide; PIP, peak inflation pressure; Pplat, plateau pressure; APTT, activated partial thromboplastin time; PT, prothrombin time; ACT, activated clotting time; NT-proBNP, N-terminal pro-B-type natriuretic peptide; cTnT, cardiac troponin T; MyO, myoglobin; CK-MB, creatine kinase-MB; EF, ejection fraction; VTI, velocity-time integral; LVID, left ventricular internal dimension; RVID, right ventricular internal dimension; PEEP, positive end-expiratory pressure; CPAP, continuous positive airway pressure; CRRT, continuous renal replacement therapy; IABP, intra-aortic balloon pump*.

### Blinding

Trial assistants from the allocation center responsible for randomization will be blinded to patients in both the groups. Each cardiopulmonary rehabilitation session provided by the therapists will be sheltered with a curtain. In the control group, an optimization procedure will be imitated as closely as possible, while no cardiopulmonary rehabilitation will be provided. This action may be helpful to blind the clinicians and nurses and mitigate any bias. Assessors, data analysts, and statisticians will be blinded to group allocation.

### Data Management and Monitoring

All the data will be entered into standardized electronic case report forms (eCRFs) and stored in a bespoke trial cloud database upon its collection. Data entry will be independently performed and dated by two trial assistants. Typos and missing data will be detected by this dual-track data entry mode. When discrepancies occur, consensus will be achieved by raw-data review or discussion. Confidentiality of data is assured by restricted access to the cloud database granted to authorized investigators only, for example, members of the Data and Safety Monitoring Committee (DSMC). The primary investigators, trial coordinators, the DSMC members, data analysts, statisticians, and trial assistants will meet periodically to: (1) monitor and review safety of patient; (2) request and perform interim data analyses; (3) review patient recruitment, accrual, and withdrawal; (4) discuss about continuing or modifying the trial; and (5) stop the trial upon any severe adverse events considered to have been caused by the CaRe-ECMO program (37).

### Sample Size Calculation

We considered the rate of ready for ECMO weaning being conservatively 50% higher in the CaRe-ECMO group than in the control group at CaRe-ECMO day 7 as a clinically meaningful effect, implying a hazard ratio (HR) of 0.5. Accordingly, we performed sample size calculation for the Cox proportional hazards model with an alpha error of 5% and a power of 80%, effect size of HR = 0.5, and overall event (ready for weaning) probability of 30% at CaRe-ECMO day 7 (increases from 30 to 45%) according to our retrospective data analysis and literature review (38). The needed sample size was estimated as *n* = 348, i.e., 174 per group. Due to the relative short observational period and the real-world situation (e.g., patients treated with ECMO therapy generally will not discharge till stable or death), accounting for 5% attrition according to their failing for fulfill of daily eligibility screening for cardiopulmonary rehabilitation during the therapeutic period, the target sample size is set at 366 (183 per group).

### Statistical Methods

The demographic and clinical characteristics collected at randomization will be presented as means with SDs or medians with interquartile ranges for continuous variables and as percentages for categorical variables, as appropriate.

For all the outcomes, we will estimate differences in effect size between the groups on both the intention-to-treat (ITT) and per protocol (PP) basis (39). Categorical variables will be compared with the chi-square or Fisher's exact tests and continuous variables will be compared with the Student's *t*-test or the Wilcoxon signed-rank test, as appropriate. The time effect of day the time-to-event outcomes identified (e.g., rate of ready for ECMO weaning, rate of ECMO weaning, rate of mechanical ventilation weaning, all-cause mortality, and rate of major post-ECMO complications) between the groups will be estimated with the Cox proportional hazards regression analysis and graphically illustrated using the Kaplan–Meier methods until CaRe-ECMO days 7, 14, 30, and 90, respectively (40). Variables, including total length of ready for ECMO weaning, total length of ECMO weaning, total length of mechanical ventilation, ECMO unit LOS, and total hospital LOS, will be treated with time-to-event data and will be analyzed with mixed-effects ordered logistic regression (41). Secondary outcomes with normal and non-normal distributions, including CPC, ADL, HRQoL, and diaphragmatic thickness and mobility, will be estimated for group differences with mixed-effects linear regression (41).

Considering that part of patients will definitely not reach the primary endpoint, i.e., ready for ECMO weaning at CaRe-ECMO day 7 for sensitivity purpose, we will assess the heterogeneity of the treatment effect on primary outcome and secondary outcomes in pre-specified subgroups of interest as per baseline data to be collected at randomization (e.g., age, gender, main diagnosis, comorbidities, the APACHE II score, ECMO mode, length since mechanical ventilation) by using exploratory *post-hoc* sensitivity adjusted analyses for any corresponding statistical methods mentioned above.

Interim analysis will be scheduled halfway through enrollment to check that there are no serious issues with respect to sample handling or data collection.

All the analyses will be conducted at a two-sided alpha level of 5% and will be performed using the Stata version 16 (StataCorp, College Station, Texas, USA) or other software, as appropriate.

## Discussion

Cardiopulmonary rehabilitation was proven to be effective in the improvement of cardiovascular and respiratory function in various major diseases (42). However, its efficacy in ECMO-supported patients has not been well-verified. Its application in routine clinical practice always encounters a number of challenges. Specifically, one of the major barriers is the multiple sites of cannulation, which restrict the mobilization of the body. This explained why most of the trials involved patients with VV-ECMO with cannulation sites in the upper body allowing for more chances of mobilization while less on VA-ECMO (4). Nonetheless, active cardiopulmonary rehabilitation is ever difficult to be performed for various reasons. In the emergency room, patients would always be sedated with the application of nitric oxide and neuromuscular blocking agents to promote oxygenation and improve ventilator synchrony. That is why, only few studies reported results with limited sample size in awake patients with ECMO (43, 44). Furthermore, active mobilization of the body may interrupt the ECMO flow (45). Therefore, the upcoming challenge is to explore a cardiopulmonary rehabilitation protocol, which can be safely delivered with no interruption of the routine clinical treatment and subsequently benefit all the types of ECMO-supported patients.

Despite the fact that most of cardiopulmonary rehabilitation carried out in the ICU and emergency room are low intensity, intensive rehabilitation has not been found to be superior to rehabilitation with relatively low intensity (46, 47). In addition, various types of cardiopulmonary rehabilitation have been documented as functional beneficial in the literature. For instance, prone positioning has been demonstrated to decrease mortality at day 28 (16.0 in the prone group vs. 32.8% in the supine group, *p*< 0.001) and day 90 (23.6 vs. 41.0%, *p*< 0.001) in patients with acute respiratory distress syndrome. Its application was strongly encouraged by several well-designed studies (27, 48). Physiotherapy such as NMES-induced muscle contraction may serve as muscle pump to increase ejection capacity and improve both the venous return and muscle perfusion, consequently compensate cardiac function (49). Moreover, ICU-acquired weakness was frequently observed in the ICU and diaphragm dysfunction developed more often than limb muscle weakness (50). Diaphragmatic weakness, occurs in as early as 18 h after mechanical ventilation, is attributed to proteolysis-induced muscle contractile dysfunction (51). It was proven to be associated with a higher rate of mechanical ventilation weaning failure and prolonged length of mechanical ventilation (52). Upon this condition, we also included electrical stimulation of phrenic nerve to minimize the reduction of diaphragm atrophy and strength over time, possibly contributing to speed up respiratory functional recovery and subsequently leading to increased rate of ECMO weaning and shortened length of ECMO therapy (53–55). Taken together, it is reasonable to hypothesize that our protocolized cardiopulmonary rehabilitation may contribute to internal pathophysiological changes (e.g., cardiac output and maximal oxygen uptake) and spread it to external clinical outcomes (e.g., earlier weaning of ECMO and increased rated of ECMO weaning).

Additional important considerations, which we addressed when preparing the CaRe-ECMO trial protocol, were the following. First, the current trial is consisting of a RCT part followed by an open-label cohort part. According to our clinical experience and retrospective data analysis, the median length of ECMO weaning varied ~from 7 to 9 days in all the types of ECMO-supported patients. The design rationale of the RCT part is to power the primary outcome, while the following open-label cohort part is dedicated to benefit patients who do not respond to one cycle of cardiopulmonary rehabilitation. This action may impair the effects of RCT on long-term outcome observation; however, it is hypothesized to provide additional opportunity for those who are probably benefitted from active intervention (56). Second, a critical concern of the CaRe-ECMO trial design is the uncertainty of the effect size of the proposed interventions. Therefore, our attention on primary outcome selection has been shifted from the exact ECMO weaning to ready for ECMO weaning. If any significant results would be introduced in terms of earlier ready for ECMO weaning or increased rate of ready for ECMO weaning due to our proposed interventions, then it may satisfy the preliminary requirements when extending its effects to more consolidated outcomes (e.g., rate of ECMO weaning). To compensate the aforementioned risks, we introduce several evidence-based checklists to ensure the standardized definition and evaluation of ECMO or mechanical ventilation weaning (Tables 5–7) and to maximally avoid potential performance bias. Third, functional outcomes are essential to the long-term recovery; therefore, CPC index, ADL score, HRQoL score, and diaphragmatic thickness and mobility will be consecutively collected. In case when primary outcome of the CaRe-ECMO trial does not show a statistically significant difference, future trials may consider to analyze our secondary clinical and functional outcomes.

To sum up, the currently proposed CaRe-ECMO trial is a pragmatic, multidisciplinary, randomized controlled, parallel group, clinical trial, designed to answer the question whether cardiopulmonary rehabilitation can facilitate weaning of ECMO. Should the implementation of the CaRe-ECMO program result in superior primary and secondary outcomes as compared to the controls, the CaRe-ECMO trial will offer an innovative treatment option for ECMO-supported patients and meaningfully impact the standard care in ECMO therapy.

## Data Availability Statement

The original contributions presented in the study are included in the article/supplementary material, further inquiries can be directed to the corresponding author/s.

## Ethics Statement

The studies involving human participants were reviewed and approved by the First Affiliated Hospital of Nanjing Medical University (Reference number of 2021-SR-416). The patients/participants provided their written informed consent to participate in this study.

## Author Contributions

XL, JZ, XC, YZ, and HS conceived the design of the trial, prepared and drafted the study protocol, and planned the statistical analysis. XL and JZ contributed to critical revision of the protocol, training of medical staff, and coordinating the trial. XC will conduct the eligibility screening for trial enrollment. YC, DP, LW, XZ, and SW are responsible for cardiopulmonary rehabilitation delivery. YM, JL, DH, FS, WL, GZ, and HZ are the clinical members of our ECMO therapy group and will be responsible for the medical care for ECMO-supported patients. YG is the leading nurse in the emergency room and will be responsible for coordination of providing nursing care for ECMO-supported patients. YZ and HS are responsible for data management and the communication with the DSMC. ZZ and BL are responsible for data acquisition and assist trial coordination. All the authors read and contributed intellectually important content and approved the final version of the manuscript.

## Funding

This trial was funded by the Nanjing Municipal Science and Technology Bureau (Grant No. 2019060002) and the National Natural Science Foundation of China (Grant Nos. 81772441, 81902288, and 82072546).

## Conflict of Interest

The authors declare that the research was conducted in the absence of any commercial or financial relationships that could be construed as a potential conflict of interest.

## Publisher's Note

All claims expressed in this article are solely those of the authors and do not necessarily represent those of their affiliated organizations, or those of the publisher, the editors and the reviewers. Any product that may be evaluated in this article, or claim that may be made by its manufacturer, is not guaranteed or endorsed by the publisher.
